# Treatment of Pruritus With Maralixibat in Early-Stage Chronic Graft Dysfunction of a Child With Alagille Syndrome

**DOI:** 10.7759/cureus.88529

**Published:** 2025-07-22

**Authors:** Rainer Ganschow, Alexander Weigert, David Katzer

**Affiliations:** 1 Department of Pediatrics, University Hospital Bonn, Bonn, DEU

**Keywords:** alagille syndrome, cholestatic pruritus, early-stage chronic graft dysfunction, ileal bile acid transporter inhibitor, liver transplant

## Abstract

Alagille syndrome (ALGS) is a rare, autosomal dominant disorder characterized by cholestasis and progressive liver disease that can lead to liver transplantation or death. Liver transplant recipients with ALGS can face chronic graft dysfunction and refractory pruritus that significantly impairs quality of life. We present the case of a 16-year-old male patient with ALGS who developed severe pruritus 13 years after receiving a liver transplant. Despite conventional therapies, his symptoms persisted, accompanied by elevated serum bile acids. Treatment with maralixibat, an ileal bile acid transporter inhibitor, resulted in complete resolution of pruritus within two months as well as a substantial reduction in serum bile acids. No adverse events were reported. This case offers real-world evidence of the effectiveness of maralixibat in managing cholestatic pruritus in a patient with ALGS and early-stage chronic graft dysfunction, highlighting its potential as a therapeutic option in this challenging patient population.

## Introduction

Alagille syndrome (ALGS) is a rare, debilitating, autosomal dominant disorder that presents with cholestasis, failure to thrive, xanthomas, and progressive liver disease and can lead to liver transplantation or death [1]. Cholestatic pruritus is the most burdensome manifestation of ALGS and is one of the most severe forms of pruritus among cholestatic liver diseases [2]. Indeed, treatment-refractory pruritus is an indication for liver transplantation in 69% to 82% of patients with ALGS [3,4]. Most patients with ALGS require liver transplantation before reaching adulthood, with transplant-free survival rates of 24% to 40% by approximately 18 years of age [4,5]. However, up to 12% of patients with ALGS who have had a liver transplant will experience graft failure within five years [4]. Graft dysfunction may be classified as early stage based on the onset of pruritus, an increase in sBA levels to over 100 µmol/L, and the presence of mild splenomegaly, which is considered an early sign of portal hypertension.

Maralixibat is a minimally absorbed ileal bile acid transporter inhibitor that prevents enterohepatic bile acid recirculation and is approved for the treatment of cholestatic pruritus in patients with ALGS aged two months or older in the EU and in patients aged three months or older in the US [6,7]. Maralixibat is also approved for the treatment of progressive familial intrahepatic cholestasis (PFIC) in patients aged three months or older in the EU and for the treatment of cholestatic pruritus in patients with PFIC aged 12 months or older in the US [6,7]. In a phase 2b study, participants with ALGS who were treated with maralixibat demonstrated significant improvements in pruritus and serum bile acid (sBA) levels [8]. In a retrospective analysis comparing maralixibat clinical trials with a natural history cohort of patients with ALGS, participants treated with maralixibat had statistically significant improvements in event-free survival (*P*<0.0001) and transplant-free survival (*P*<0.0001) compared with the natural history cohort [9]. However, there are limited data on the use of maralixibat for managing cholestatic pruritus in patients with ALGS who experience graft failure after a liver transplant.

The objective of this report is to demonstrate real-world experience with maralixibat to treat cholestatic pruritus in a patient with ALGS 13 years after receiving a liver transplant.

This case was presented in part as a poster at the 2023 Gesellschaft für Pädiatrische Gastroenterologie und Ernährung (GPGE) Annual Conference, March 22-25, 2023, Stuttgart, Baden-Württemberg, Germany.

## Case presentation

**Figure 1:**
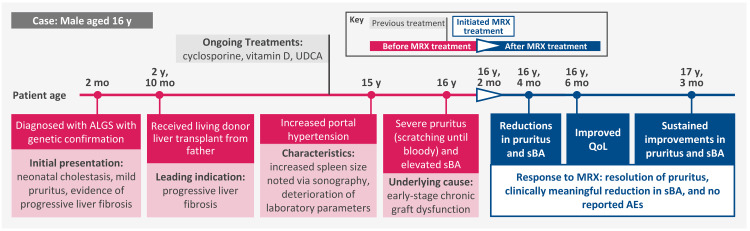
Clinical History Timeline The white triangle indicates the initiation of maralixibat treatment. Pink boxes indicate clinical events before maralixibat treatment. Blue boxes indicate clinical events after maralixibat treatment. AE, adverse event; ALGS, Alagille syndrome; MRX, maralixibat; QoL, quality of life; sBA, serum bile acid; UDCA, ursodeoxycholic acid.

The patient is a 16-year-old boy who was diagnosed with ALGS, with genetic confirmation, at two months of age (Figure 1). At diagnosis, the patient presented with neonatal cholestasis and mild pruritus, with a score of 1 on the Itch-Reported Outcome (Observer) (ItchRO[Obs]) scale (range, 0-4). He also presented with evidence of progressive liver fibrosis that was the leading indication for his eventual liver transplant. At age two years, 10 months, the patient received a living donor liver transplant from his father. The patient had no clinical complications in the years that followed and received cyclosporine for immunosuppression, vitamin D, and ursodeoxycholic acid. He exhibited no other organ dysfunction, such as cardiac or renal complications. The marked hypercholesterolemia present before liver transplantation resolved quickly after the procedure.

**Figure 2:**
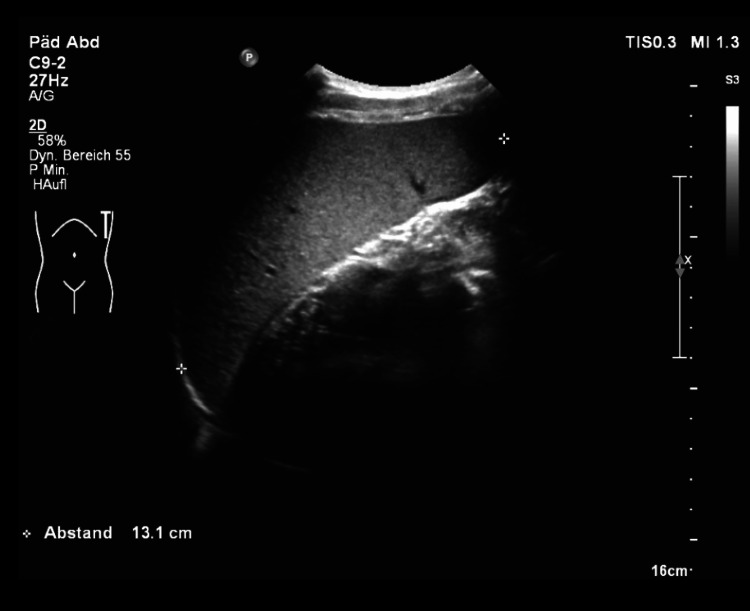
Ultrasonography of the Abdomen Showing a Slightly Enlarged Spleen Before maralixibat treatment, the spleen measured 13.1 cm (normal age-related value, 9.5–12.5 cm).

**Figure 3:**
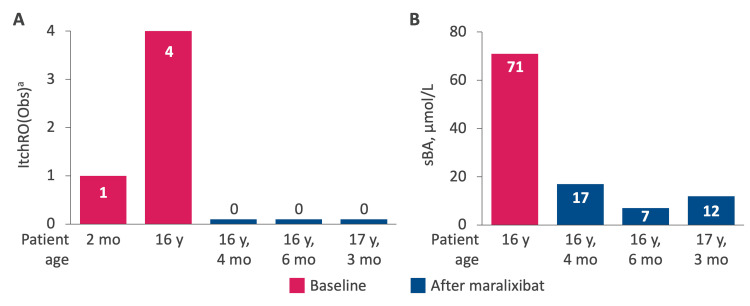
ItchRO(Obs) Score and Serum Bile Acid Levels Before and After Treatment With Maralixibat (A) ItchRO(Obs) score over time and (B) sBA levels (µmol/L) over time. Pink bars represent timepoints before maralixibat treatment. Blue bars represent timepoints after maralixibat treatment. ^a^ItchRO(Obs) is a 0 to 4 scale, where 0 = no itch, 1 = mild, 2 = moderate, 3 = severe, and 4 = very severe; a ≥1-point reduction is considered clinically meaningful [8]. ItchRO(Obs), Itch-Reported Outcome (Observer); sBA, serum bile acid.

Twelve years after the transplant, the patient developed increased portal hypertension characterized by an increase in the size of the spleen detected via sonography and deterioration of laboratory parameters (Figure 2, Table 1). Splenomegaly was mild (13.2 cm), and the size of the spleen prior to transplant was within the reference range (12 cm; reference range for patient age, 9.5-12.5 cm). At this time, alanine aminotransferase (ALT) was 16 U/L, aspartate aminotransferase (AST) was 18 U/L, gamma-glutamyl transferase (GGT) was 14 U/L, and total bilirubin was 1.2 mg/dL. Beginning just over 13 years after liver transplantation, the patient developed early-stage chronic graft dysfunction marked by severe pruritus (ItchRO[Obs] score = 4) and scratching himself until bloody (Figure 3A). The patient also had elevated sBA levels (71 µmol/L; Figure 3B). Treatment with maralixibat was initiated at age 16 years, two months.

**Table 1:** Laboratory Assessment Values at Baseline and After Maralixibat Treatment ALT, alanine aminotransferase; AST, aspartate aminotransferase; GGT, gamma-glutamyl transferase; NR, not reported. *Reported reference ranges for laboratory parameters are specific to Germany.

Laboratory assessment	Reference ranges^*^	Baseline (age 16 y)	After maralixibat treatment		
			2 mo	4 mo	13 mo
ALT, U/L	5-39	16	18	17	26
AST, U/L	15-53	18	19	14	27
GGT, U/L	5-17	14	16	NR	18
Total bilirubin, mg/dL	0-0.9	1.2	1.2	1.1	1.4
Direct bilirubin, mg/dL	< 0.3	NR	0.3	NR	NR

Two months after maralixibat initiation, the patient had complete resolution of pruritus (ItchRO[Obs] score = 0) and clinically meaningful reductions in sBA levels (17 µmol/L; Δ: −54 µmol/L). At this time, ALT was 18 U/L, AST was 19 U/L, GGT was 16 U/L, and total bilirubin was 1.2 mg/dL. After four months of treatment with maralixibat, sBA levels were further reduced to 7 µmol/L, the ItchRO(Obs) score was 0, and the patient had dramatically improved quality of life. After 13 months of treatment with maralixibat, sBA levels were 12 µmol/L, and the patient had no pruritus and slightly soft stools that were tolerable. Overall, maralixibat was well tolerated, and no adverse events were reported.

## Discussion

This case provides the first real-world evidence illustrating the effective use of maralixibat for managing cholestatic pruritus that developed due to early-stage chronic graft dysfunction 13 years after liver transplantation in a patient with ALGS. The patient’s history of neonatal cholestasis, progressive liver disease, and eventual liver transplantation aligns with the common clinical presentation of ALGS; however, there is a wide range of clinical manifestations of ALGS [1]. Despite the success of the transplant, the development of early-stage chronic graft dysfunction and worsening pruritus highlight the ongoing challenges faced by patients with ALGS, even after transplantation.

The patient’s presentation with severe pruritus and elevated sBA levels, both hallmarks of cholestasis, is consistent with the pathophysiological mechanisms in ALGS, where impaired bile acid transport due to bile duct paucity leads to bile acid accumulation and pruritus [1,2]. Furthermore, the reduction in sBA levels from 71 µmol/L to 17 µmol/L after two months of maralixibat treatment in this patient suggests that maralixibat effectively interrupts the enterohepatic recirculation of bile acids, thus decreasing their toxic accumulation in the liver and improving cholestasis [8]. These results are consistent with those in the phase 2b ICONIC study, which demonstrated reductions in sBA levels and improvements in pruritus in participants with ALGS who received maralixibat [8].

The reduction in pruritus, with the patient’s ItchRO(Obs) score decreasing from 4 to 0, is particularly notable, as pruritus in chronic liver diseases can severely affect health-related quality of life. In an analysis of the ICONIC study, participants who had improvements in pruritus after maralixibat treatment demonstrated clinically meaningful improvement in health-related quality of life, as well as greater improvements in family impact and fatigue scores, compared with pruritus nonresponders [10]. The reduction in pruritus is also notable given that pruritus in ALGS is often refractory to conventional therapies [3,4].

While the results of this single case report are encouraging, it is possible that treatment with maralixibat may not be as effective in all patients with early-stage chronic graft dysfunction. Further research is warranted to determine the effectiveness of maralixibat in patients with ALGS who experience pruritus after liver transplant.

## Conclusions

In conclusion, this case demonstrates the effective use of maralixibat to treat pruritus that developed 13 years after liver transplantation in a patient with ALGS. Treatment with maralixibat was well tolerated with no adverse events reported. Although a single case study, the rapid and sustained improvements in pruritus and sBA levels observed in this patient provide real-world evidence supporting the therapeutic potential of maralixibat in patients with ALGS experiencing early-stage chronic graft dysfunction after liver transplantation. The continued follow-up of this patient will provide important information on long-term outcomes.
